# Effectiveness of video self-instruction training on cardiopulmonary resuscitation retention of knowledge and skills among nurses in north-western Nigeria

**DOI:** 10.3389/fpubh.2023.1124270

**Published:** 2023-03-21

**Authors:** Ahmed Saidu, Khuan Lee, Iskasymar Ismail, Oyedunni Arulogun, Poh Ying Lim

**Affiliations:** ^1^Department of Nursing, Faculty of Medicine and Health Sciences, Universiti Putra Malaysia, Serdang, Selangor, Malaysia; ^2^Federal University Birnin-Kebbi, Birnin Kebbi, Kebbi, Nigeria; ^3^Department of Medicine, Faculty of Medicine and Health Sciences, Universiti Putra Malaysia, Serdang, Selangor, Malaysia; ^4^RESQ Stroke Emergency Unit, Hospital Sultan Abdul Aziz Shah, Universiti Putra Malaysia, Serdang, Malaysia; ^5^Department of Health Promotion and Education, Faculty of Public Health, College of Medicine, University of Ibadan, Ibadan, Nigeria; ^6^Department of Community Health, Faculty of Medicine and Health Sciences, Universiti Putra Malaysia, Serdang, Selangor, Malaysia

**Keywords:** Nigeria, cardiopulmonary resuscitation, nurses, knowledge, skills, resuscitation, intervention

## Abstract

**Background:**

Adaptable cardiopulmonary resuscitation/basic life support (CPR/BLS) training are required to reduce cardiac arrest mortality globally, especially among nurses. Thus, this study aims to compared CPR knowledge and skills retention level between instructor-led (control group) and video self-instruction training (intervention group) among nurses in northwestern Nigeria.

**Methods:**

A two-arm randomized controlled trial study using double blinding method was conducted with 150 nurses from two referral hospitals. Stratified simple random method was used to choose eligible nurses. For video self-instruction training (intervention group), participants learnt the CPR training *via* computer in a simulation lab for 7 days, in their own available time whereas for instructor-led training (control group), a 1-day program was conducted by AHA certified instructors. A generalized estimated equation model was used for statistical analysis.

**Results:**

Generalized Estimated Equation showed that there were no significant differences between the intervention group (*p* = 0.055) and control group (*p* = 0.121) for both CPR knowledge and skills levels respectively, whereas higher probability of having good knowledge and skills in a post-test, one month and three-month follow-up compared to baseline respectively, adjusted with covariates (*p* < 0.05). Participants had a lower probability of having good skills at 6-month follow-up compared to baseline, adjusted with covariates (*p* = 0.003).

**Conclusion:**

This study showed no significant differences between the two training methods, hence video self-instruction training is suggested can train more nurses in a less cost-effective manner to maximize resource utilization and quality nursing care. It is suggested to be used to improve knowledge and skills among nurses to ensure cardiac arrest patients receive excellent resuscitation care.

## 1. Introduction

The purpose of the cardiopulmonary resuscitation/basic life support (CPR/BLS) instruction program is to equip healthcare providers, including laypeople, with the knowledge and skills needed to manage cardiac arrest emergencies until advanced medical support appears (1). As a result, nurses are to be familiar with CPR and be able to perform it in the event of a cardiac arrest (2). Instructor-led CPR training, also known as traditional training, is the most common type of training taught to staff and laypersons. Regrettably, knowledge and skills gained through this method were found to decline more quickly, sometimes as early as 3 months, indicating that learners did not achieve the desired training outcome (3, 4). Undoubtedly, instructor-led CPR training is a highly resource-intensive program that consumes more time, manpower, and space, thus serving as a barrier to effective learning among the healthcare workers.

The limitation of these current practices and the need to train and re-train more healthcare providers and laypeople necessitated the International Liaison Committee on Resuscitation (ILCOR), through its task force, the Education Implementation and Team (EIT), to advocate the use of the video self-instruction training method as an alternative to the traditional instructor-led BLS training more than a decade ago (5, 6). This method can be an alternative option for training more healthcare providers at a low cost as well as for those who prefer to learn at their own pace (7).

In spite of the fact that previous studies had examined the effectiveness of these two training approaches, their findings reported mixed results on the acquisition and retention of CPR knowledge and skills. For instance, Hernández-Padilla et al. (3) showed a success rate among participants in the self-directed training approaches after a 3-month intervention as compared to the instructor-led method (*p* < 0.001). Similarly, Saiboon et al. (8) discovered that nurses used the self-instructed video approach demonstrated superior knowledge acquisition and retention compared to the instructor-led group (*p* = 0.03). Another two studies found no significant differences in knowledge and skill competencies (9, 10).

Unlike in many industrialized countries, professional CPR/BLS training is not a requirement for continuously certifying hospitals in many developing countries or even a prerequisite for employing healthcare workers including nurses (11, 12). This has therefore created a disparity of CPR knowledge and skill as well as competencies in identifying and treating cardiac arrest patients under their care among healthcare staff in many African countries (13, 14).

In Nigeria, there are paucity studies conducted to determine whether video self-instruction CPR training has the same impact as instructor-led training on knowledge and skill. Till now, instructor-led training has been the most popular training method in Nigeria, both in training schools and in hospital settings. This study aims to add to the body of literature by comparing the effectiveness of a video self-instruction training method with. instructor-led CPR on knowledge and skill retention at pre-test, post-test, and follow-up at 1 month, 3 months, and 6 months among hospital nurses in north-western Nigeria.

## 2. Materials and methods

### 2.1. Study design

A two-arm randomized controlled trial (RCT) was conducted to assess the effectiveness of two CPR training methods among nurses in two out of seven selected hospitals in north-western Nigeria namely, Kano, and Sokoto states. A detailed explanation of the protocol is listed in “Effectiveness of cardiopulmonary resuscitation (CPR) using video self-instruction training on the level of knowledge and skills retention among hospitals nurses in north-western Nigeria-Protocol paper”, The study was implemented among 150 nurses between July 2021 to January 2022.

### 2.2. Sampling and population

A stratified simple random selection method was used to choose eligible nurses from the different wards/units of the identified two referral hospitals. The hospital wards were numbered from 1 to N based on a ward/unit duty roster list of all registered nurses from the Head of Department nursing office in each of the two referral hospitals. Each stratum (ward/unit) was allocated a computer-generated random number, with the size according to the staff makeup. Inclusion criteria included being a registered nurse with the Nigerian Nursing Council, and have given their written consent, being able to read and write in English and are currently working in the following wards/unit; male and female medical, and surgical wards, as well as intensive care unit (ICU), accident and emergency (A&E) and general outpatient department (GOPD). The exclusion criteria included those that were be trained in CPR in the last 3 months before the start of this study.

The sample size was computed using two mean formula (15), with the mean ± SD score of CPR knowledge at pretest among the instructor-led group (11.37 ± 2.70) and posttest (125.95 ± 2.26), 95% confidence level, 80% power, 70% response rate, and 1.3 design effect. The final sample size was 150, with 75 nurses recruited from every two hospitals as reported in one of the CPR studies (8). Double blinding method was adopted, where neither the participants nor the AHA instructor who assess both knowledge and skills was aware of the randomization group.

### 2.3. Procedure

The training method employed in this study was the video self-instruction method as (intervention group) which was an interactive learning activity for healthcare professionals that uses a BLS HeartCode DVD, while the instructor-led (control group) training had two instructors used the traditional lectures, presentations, demonstrations, and discussions methods.

#### 2.3.1. Intervention group

**Figure 1:**
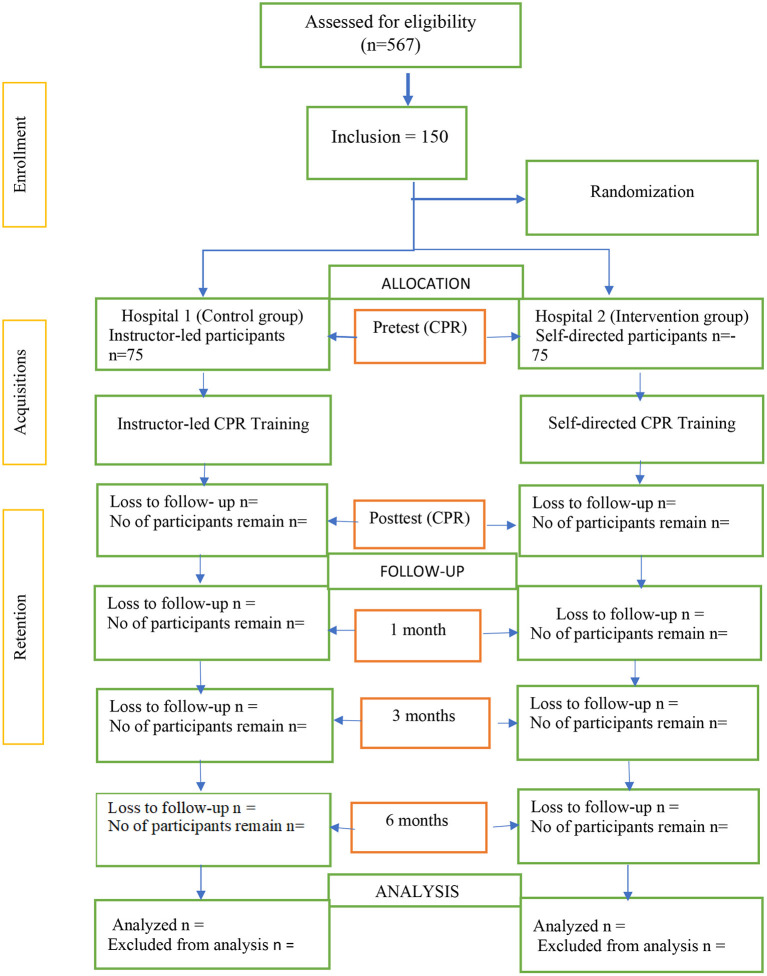
Flow-chart. Flow chart according to CONSORT (www.consort-statement.org) to compare the effectiveness of CPR/BLS training between video self-instructor and instructor-led training.

Participants for the intervention group had BLS HeartCode CD installed on a computer, a screen projector and Laerdal Resusci Anne that is without performance feedback to the learners during practice situated in a simulation lab within the hospital premises. Each participant also received a self-prepared manual with learning objectives and guidelines for learning. The training duration lasted for only 7 days and was done in the participants' leisure time. Participants learn through all the CPR knowledge and skills sections on their own and can choose how and where to start their learning activities using the video player's rewind, forward, and pause controls. A research assistant was only there to assist with the setting up of the manikins, Automated External Defibrillator machine and other administrative tasks. An immediate post-test evaluation was conducted, thereafter participants have no access to the DVD and the manikins until the next follow-up test at 1-, 3-, and 6 months. Figure 1 shows the study design according to the Consolidated Standards of Reporting Trials (CONSORT) Statement.

#### 2.3.2. Control group

Participants in the control group received a CPR manual which included learning objectives adapted from the AHA healthcare providers manual. Two AHA-certified instructors provided training to the group of trainees to learn BLS/CPR knowledge and skills using standardized traditional classroom instruction. This was a 1-day program that lasted for 7 h including the pretest, with one instructor for every 20–30 nurses due to resource-limited training materials. A post-test was administered at the conclusion of the training and the subsequent 1-, 3-, and 6 months follow-up.

### 2.4. Data collection

The instruments for the data collection method were both administering questionnaires and the checklist for skills tests using the manikins. The self-administrated questionnaire was adapted from a previous study with 3 sections, where the first section was socio-demographic data of the nurses as well as a profile of their working experiences; second section assessed the CPR knowledge level with 25 closed-ended multiple-choice questions (MCQs) adapted from the AHA manual for healthcare providers with only four options (A to D) (16); third section was the skill checklist instruments that assessed participants' skills competencies by AHA-certified BLS instructors using the Berden and Graham and Collin scoring system. Three scores for skills scoring system, where scored “0” for participants did correctly, “5” was scored for the competencies done wrongly and “10” for those competencies that did not perform (17, 18). Total scores range from 0 to 100. Therefore, participants that scored <10 were regarded as good skills, while those who scored 11 and above were considered to have poor skills (17, 18). The content validity of the questionnaire was performed by experts and a pilot study was conducted among 15 participants. They filled out a face validity form and a total of 93.3% agreed that all the questions were satisfactory. The Cronbach's α reliability value for knowledge questionnaire was 0.708 and considered acceptable reliable questionnaire. AHA instructors administered questionnaires and skills checklist to each participant in the intervention and control groups who had received training in five sequences: pretest before training, posttest immediately after training (7-day intervention and 7 h control group), then followed by one, 3 and 6-months follow-ups. The same AHA instructor conducted skills evaluation for five sessions to reduce the inter-rater bias.

### 2.5. Ethical consideration

Ethical approval was received from Ethic Committee For Research Involving Human Subject, Universiti Putra Malaysia (JKEUPM-2020-185) as well as two hospitals). Each participant gave informed consent before participation. All the study data was handled with strict confidence and privacy. All participants can stop participating in the study, and their rights cannot be unaffected.

### 2.6. Data analysis

Descriptive analysis was conducted where frequency and percentages were used to present the categorical data. Chi-square test and fisher exact test were be used to investigate the differences of categorical variables between intervention and control group at baseline.

The Generalized Estimating Equation (GEE) model using the Statistical Program for the Social Sciences (SPSS) version 27.0 was used for comparing the differences in knowledge and skills between the intervention and control groups across the time points, adjusted with covariates. Variable with p <0.05 was considered statistically significant. Intention-to-treat (ITT) was used in the analysis to deal with the missing data. The last Observation Carried Forward (LOCF) method was used to impute the missing data (19, 20).

## 3. Results

### 3.1. Response rate

The response rate of both the intervention and control groups at the pretest and posttest was 100% among the participants. However, at the end of 6 months, the retention rate for intervention group and the control group were 94.7% and 93.3%, respectively.

### 3.2. Sociodemographic and work-related factors of nurses between the intervention and control group at baseline (*N* = 150)

Table 1 demonstrates the sociodemographic and work-related factors at baseline among the groups revealed that only six factors were significantly difference between the intervention and control groups, using chi square test. Gender (χ2 = 7.407, *p* = 0.006), age group (χ2 = 16.071, *p* < 0.001), professional status (χ2 = 19.231, *p* < 0.001), year of work experience group (χ2 = 16.524, *p* < 0.001), duration of CPR training group (χ2 = 17.404, *p* < 0.001), and patient survival outcome (χ2 = 9.843, *p* = 0.007) were significant difference between both intervention group and control group. The significant variables were be adjusted in the final model as confounders.

**Table 1:** Baseline comparison of sociodemographic and work-related factors between the intervention and control groups (*N* = 150).

**Variables**	**Intervention (*****n =*** **75)**		**Control (*****n =*** **75)**		**χ^2^**	***P*** **-value**
	***n***	**%**	***n***	**%**		
**Gender**						
Men	19	25.3	35	46.7	7.407^a^	0.006^*^
Women	56	74.7	40	53.3		
**Age (years) group**						
20–49	54	72.0	72	96.0	16.071^a^	<0.001^*^
50 and above	21	28.0	3	4.0		
**Professional status**						
RN	30	40.0	22	29.3	19.231^a^	<0.001^*^
RN/RM	45	60.0	36	48.0		
RN/Others	0	0.0	17	22.7		
**Academic qualification group**						
Diploma in Nursing	17	22.7	26	34.7	3.628^a^	0.163
Diploma in Nursing/ Midwife	24	32.0	25	33.3		
BSc/Masters/PhD in Nursing	34	45.3	24	32.0		
**Years of work experience group**						
1–10years	9	12.0	31	41.3	16.524^a^	<0.001^*^
11–30years	62	82.7	41	54.7		
31 and above	4	5.3	3	4.0		
**Current area of work group**						
Critical area	23	30.7	33	44.0	2.850^a^	0.091
Non-critical area	52	69.3	42	56.0		
**Formal training in CPR**						
No	45	54.0	63	84.0	1.012^a^	0.314
Yes	30	21.0	12	16.0		
**Duration of CPR training group**						
Not train	41	54.7	63	84.0	17.404^a^	<0.001^*^
<5years	13	17.3	8	10.7		
≥5years	21	28.0	4	5.3		
**BLS training**						
No	54	72.0	60	80.0	1.316^a^	0.251
Yes	21	28.0	15	20.0		
**ACLS training**						
No	60	80.0	73	97.3	1.430^a^	0.232
Yes	15	20.0	2	2.7		
**BPLS training**						
No	69	92.0	70	93.3	0.098^a^	0.754
Yes	6	8.0	5	6.7		
**Frequency of CPR performance group**						
Never	4	5.3	5	6.7	0.118^a^	0.731
Yes	71	94.7	70	93.3		
**Survival of outcome**						
None survive	54	72.0	43	57.3	9.843^a^	0.007^*^
Some survive	14	18.7	30	40.0		
All survive	7	9.3	2	2.7		

* Variables with p < 0.05.

a Chi square test value.

### 3.3. Effectiveness of video self-instruction training on CPR knowledge level retention across time points (pretest, posttest, 1 month, 3 months, and 6 months) adjusted with covariates

Table 2 compares the effectiveness of video self-instruction training (intervention group) and instructor-led training (control group) on CPR knowledge level, adjusted with covariates. There were no significant differences in the probability of having a good knowledge level between the two groups (AOR = 2.065, 95%CI: 0.985, 4.328, *p* = 0.055). Participants at the posttest (AOR = 80.885, 95%CI: 17.735, 368.891, *p* < 0.001), 1-month follow-up (AOR = 7.252, 95%CI: 3.497, 15.039, *p* < 0.001), and three-month follow up (AOR = 3.227, 95%CI: 1.600, 6.505, *p* < 0.001) had higher probability of having good knowledge than at the baseline, adjusted for covariates whereas there was no significant improvement in having a higher knowledge level at 6 month among intervention group compared to baseline (AOR=0.518, 95% CI: 0.239, 1.122, *p* = 0.095). The interaction between time and group was not significant in the final model.

**Table 2:** Effectiveness of video self-instruction (intervention group) and instructor-led (control group) on CPR knowledge level across time points (pretest, posttest, 1 month, 3 month, and 6 month), adjusted with covariates.

**Variables**	**Adjusted coefficient**	**Standard error**	**Adjusted odds ratio**	**95% Cl for odds ratio**		***P*** **-value**
				**Lower bound**	**Upper bound**	
Intercept	−0.180	0.341		
**Group**						
Control	Ref			
Intervention	0.725	0.378	2.065	0.985	4.328	0.055
**Time point**						
Pretest (Baseline)	Ref			
Posttest	4.393	0.774	80.885	17.735	368.891	<0.001^*^
1 month	1.981	0.372	7.252	3.497	15.039	<0.001^*^
3 months	1.171	0.358	3.227	1.600	6.505	0.001^*^
6 months	−0.657	0.394	0.518	0.239	1.122	0.095
**Interaction**						
Control ^*^ pretest (baseline)	Ref			
Intervention ^*^ posttest	−0.904	1.030	0.405	0.054	3.050	0.380
Intervention ^*^ 1 month	0.217	0.568	1.243	0.408	3.783	0.702
Intervention ^*^ 3 month	−0.594	0.505	0.552	0.205	1.485	0.239
Intervention ^*^ 6 month	0.134	0.525	1.143	0.409	3.198	0.798

* Variable with p < 0.05.

SE, standard error; CI, Confidence interval; Ref, Reference category.

QIC = 783.439, QICC = 778.062, knowledge model adjusted with covariates: gender, age (years) group, professional status, years of experience group, duration of CPR training and survival outcome.

### 3.4. Effectiveness of video self-instruction training on CPR skills retention across time points (pretest, posttest, 1 month, 3 months, and 6 months) adjusted with covariates

Table 3 compares the effectiveness of video self-instruction training (intervention group) and instructor-led training (control group) on skills retention, adjusted with covariates. As for the skills, the probability of having good skills was not significant differences between the two groups (AOR = 1.749, 95% CI: 0.863, 3.547, *p* = 0.121). Nurses at the posttest (AOR = 104.217, 95%CI: 15.124, 718.126, *p* < 0.001), 1-month follow-up (AOR = 7.346, 95%CI: 3.333, 16.190, *p* < 0.001), and 3-month follow-up (AOR = 2.745, 95%CI: 1.387, 5.431, *p* = 0.004) had higher probability of having good skills than those at the pretest (baseline) respectively, however, nurses at 6 months had lower probability of having good skills than at baseline (AOR = 0.310, 95%CI: 0.142, 0.677, *p* = 0.003). By looking into the interaction between time and group, the intervention group at posttest had lower probability of having good skills than the control group at baseline (AOR = 0.091, 95% CI: 0.010, 0.817, *p* = 0.032), after controlling for covariates.

**Table 3:** Effectiveness of video self-instruction training (intervention group) and instructor-led (control group) between intervention and control group on CPR skills retention across time points (pretest, posttest, 1 month, 3 month, and 6 month), adjusted with covariates.

**Variables**	**Adjusted coefficient**	**Standard error**	**Adjusted odds ratio**	**95% Cl for odds ratio**		***P*** **-value**
				**Lower bound**	**Upper bound**	
Intercept	−0.341	0.364		
**Group**						
Control	Ref			
Intervention	0.559	0.361	1.749	0.863	3.547	0.121
**Time point**						
Pretest (Baseline)	Ref			
Posttest	4.646	0.985	104.214	15.124	718.126	<0.001^*^
1 month	1.994	0.403	7.346	3.333	16.190	<0.001^*^
3 months	1.010	0.348	2.745	1.387	5.431	0.004^*^
6 months	−1.171	0.399	0.310	0.142	0.677	0.003^*^
**Interaction**						
Control ^*^ pretest (baseline)	Ref			
Intervention ^*^ posttest	−2.395	1.119	0.091	0.010	0.817	0.032^*^
Intervention ^*^ 1 month	−0.360	0.624	0.698	0.205	2.373	0.565
Intervention ^*^ 3 month	−0.607	0.516	0.545	0.198	1.498	0.239
Intervention ^*^ 6 month	−1.147	0.605	0.318	0.097	1.040	0.058

* Variable with p < 0.05.

SE, standard error; CI, Confidence interval; Ref, Reference category.

QIC 721.713, QICC 721.236, skill model adjusted with covariates: gender, age (years) group, professional status, years of experience group, duration of CPR training and survival outcome.

## 4. Discussion

Proficiency in CPR is a prerequisite for nurses because they are frequently the first to respond to an in-hospital cardiac arrest. Several studies assessing the effectiveness of various teaching methods have been published. This study sought to add to the existing literature by comparing the effectiveness of the video self-instruction training (intervention group) and the instructor-led training (control group) on CPR knowledge and skill retention among nurses as across time points (posttest, 1-, 3-, and 6-month follow-up periods afterwards).

After controlling for covariates, the results revealed that there were no statistically significant differences in knowledge level between two groups (*p* = 0.055). Furthermore, the interaction of video self-instruction training across time points was not statistically significant when compared to the instructor-led training at baseline. This meant that neither self-instruction training nor instructor-led learning was more effective in acquiring and retaining participants' CPR knowledge. Bylow et al. (9) reported there was no significant effects at 6 months after training, even though the participants were laypeople with a larger sample size. Another study, conducted by Saiboon et al. (8) found that after 6 months, the self-instruction group retained more knowledge than the instructor-led group (*p* = 0.003), while their performance on skills was not statistically different (*p* = 0.47). Other studies conducted in the United States by Braslow et al. (21) and Todd et al. (22) reported no statistical difference in knowledge level between the self-instruction and instructor-led groups, but the participants' skills differed considerably.

Interestingly, this study found that, after adjusting for covariates, participants were more likely to have good CPR knowledge of the two training methods at the posttest, one-, and three-month follow-ups than they were at the baseline. Previous studies by Aqel and Ahmad (23), Hernández-Padilla et al. (3), and Serwetnyk et al. (24) found that comparable increases in CPR knowledge and skill score retention between pretest and posttest, posttest and follow-up test, or pretest and follow-up (*p* < 0.001).

In terms of skill competencies, the outcomes of this study revealed that there were no statistically significant differences in skill level at the 6-month follow-up (*p* = 0.121). This outcome was consistent with a study published by Mardegan et al. (10), but it may have limitations since it only used posttest and 4-week posttest scores following training rather than the 6 months reported in the current study. According to their study's findings, neither instructor-led instruction nor self-instruction training was better for improving the two learner groups' skills to acquire and retain CPR skills. While findings of this study, however, revealed a significant difference in the acquisition of skill competencies, as evidenced by the posttest (*p* = 0.032). This could be explained by the fact that intervention group participants had access to the BLS training manual for 7 days, but control group participants only had 8 h of contact before the post-test. Despite this, previous studies reported no discernible differences between the intervention and control groups (25, 26).

Similar to knowledge level, the two learning approaches also significantly improved skill retention from baseline to posttest, 1 month, 3 months, and 6 months later, after controlling for covariates. In contrast to their knowledge level, participants after the 6-month follow-up still had lower good skills compared with baseline (p = 0.003). These findings are contrast with a previous study, which revealed that participants had high levels of competency after a 6-month CPR training course (27–29). Similarly, studies by Aliyari et al. (30), Hernández-Padilla et al. (3), and Mardegan et al. (10) revealed a significant level of skill retention, albeit for a shorter period. Other studies, such as those by Bhanji et al. (31), Cheng et al. (32), Greif et al. (33), Kardong-Edgren et al. (34), and Oermann et al. (35), support this study's findings that knowledge retention after training can only last 3–6 months. Many resuscitative investigations demonstrated that skill level decline faster than knowledge level (11, 14, 34–36). This may be related to how video self-instruction training allows students to engage in lifelong learning by actively involving them in the processes of identifying learning needs, selecting learning outcomes, implementing learning strategies, and reflecting on the development of their new competencies (37, 38). As a result, nurses must regularly receive CPR training if they expect to retain or improve their skills (30, 39, 40). This is because several studies have shown that BLS knowledge levels might decline in the absence of consistent training (36, 41).

Several implications for nursing practice are suggested, for instance video self-instruction training can eliminate some of the most significant barriers that hospital nurses and other healthcare providers face to acquiring CPR/BLS training at the appropriate time. As is well known, there aren't enough CPR instructors to teach the number of working staff who may require certification or re-certification, or even students who are unable to attend instructor-led courses and thus fail to complete their recertification requirements. As a result, video self-instruction training can be an effective substitute for instructor-led training, allowing institutions to fill in the gaps. More nurses will be trained at a reduced cost when this training approach is employed in a hospital, freeing up the CPR instructors' time to pursue other educational goals that they previously were unable to, including frequently conducting mock tests on all of the hospital staff in the wards and units.

Notwithstanding, the researcher recognizes the benefit of a new or inexperienced nurse participating in instructor-led classroom training since it fosters group interaction and provides feedback to both the teacher and the learners. However, by using video self-instruction training approaches, trainees will build confidence in the real world faster because they will know that their mistakes will not be detrimental during simulation practice.

## 5. Conclusion

In terms of acquiring and retaining knowledge and skill levels among the two groups, neither training strategy was found to be more effective than the other. However, significant improvement in acquisition and retention was observed from the posttest to 3 months compared to the baseline. Therefore, nurses must obtain regular refresher training to keep their knowledge and skills up to date, especially in places where CPR procedures are rarely performed on patients. This will improve nurse learning and retention while also ensuring that cardiac arrest patients receive optimal resuscitation care.

## Data availability statement

The datasets presented in this study can be found online at https://doi.org/10.17632/bdmbm8ny37.1. The names of the repository/repositories and accession number(s) can be found in the article/supplementary material.

## Ethics statement

The studies involving human participants were reviewed and approved by Ethics Committees and an Institutional Review Board of Universiti Putra Malaysia have authorized this study (Approval No. 2020-185). The patients/participants provided their written informed consent to participate in this study.

## Author contributions

Conceptualization and formal analysis: AS and PL. Methodology: AS, PL, KL, II, and OA. Supervision and writing—review and editing: PL, KL, II, and OA. Writing—original draft: AS. All authors contributed to the article and approved the submitted version.
